# Gene set analysis methods: a systematic comparison

**DOI:** 10.1186/s13040-018-0166-8

**Published:** 2018-05-31

**Authors:** Ravi Mathur, Daniel Rotroff, Jun Ma, Ali Shojaie, Alison Motsinger-Reif

**Affiliations:** 1 Bioinformatics Research Center, North Carolina State University, Raleigh, NC USA; 2 Department of Statistics, North Carolina State University, Raleigh, NC USA; 3 Department of Biostatistics, University of Washington, Seattle, WA USA; 4 Present address: RTI International, Research Triangle Park, Durham, NC USA

**Keywords:** Gene set analysis, Pathway analysis, Methods comparison

## Abstract

**Background:**

Gene set analysis is a valuable tool to summarize high-dimensional gene expression data in terms of biologically relevant sets. This is an active area of research and numerous gene set analysis methods have been developed. Despite this popularity, systematic comparative studies have been limited in scope.

**Methods:**

In this study we present a semi-synthetic simulation study using real datasets in order to test and compare commonly used methods.

**Results:**

A software pipeline, Flexible Algorithm for Novel Gene set Simulation (FANGS) develops simulated data based on a prostate cancer dataset where the KRAS and TGF-β pathways were differentially expressed. The FANGS software is compatible with other datasets and pathways. Comparisons of gene set analysis methods are presented for Gene Set Enrichment Analysis (GSEA), Significance Analysis of Function and Expression (SAFE), sigPathway, and Correlation Adjusted Mean RAnk (CAMERA) methods. All gene set analysis methods are tested using gene sets from the MSigDB knowledge base. The false positive rate and power are estimated and presented for comparison. Recommendations are made for the utility of the default settings of methods and each method’s sensitivity towards various effect sizes.

**Conclusions:**

The results of this study provide empirical guidance to users of gene set analysis methods. The FANGS software is available for researchers for continued methods comparisons.

**Electronic supplementary material:**

The online version of this article (10.1186/s13040-018-0166-8) contains supplementary material, which is available to authorized users.

## Introduction

Gene expression data, especially at the whole genome level, is a powerful tool in modern genomics. Microarray, and more recently, RNA-sequencing data have been leveraged to interrogate a wide variety of biological problems. [12, 17, 36, 40] Gene expression data has been collected for a wide variety of study designs, but most commonly to compare gene expression patterns across groups/classes (such as cases vs. controls or exposed vs. unexposed). Such group comparisons are performed with a number of biological goals, broadly categorized as class comparison (for example, is differential expression associated with case/control status), class prediction (for example, can gene expression data be used to predict disease), or class identification (for example, can gene expression data be used to diagnose a disease or disease subtype). While the exact details of analysis will depend on the particulars of the data and the goals, there are common workflows that have emerged.

Data analysis workflows for gene expression analysis have evolved significantly over the last 15 years, with a broad number of quality control, association tools and multiple testing control approaches developed specifically for such data. The first step of a gene expression study typically involves the evaluation of expression at the single gene level, which produces a list of associated genes ranked by the magnitude of the statistical association. While this is a crucial first step, investigators often conduct genome wide expression analysis to obtain a more global view of expression changes, and the ability to put these gene-level results into a broader biological context is highly desirable. Gene-set analysis (GSA), also referred to as pathway analysis, is a commonly used approach to address these goals. In GSA, genes are aggregated into gene sets on the basis of shared biological or functional properties as defined by a reference knowledge base. Knowledge bases are database collections of molecular knowledge which may include molecular interactions, regulation, molecular product(s) and even phenotype associations. The resultant gene sets are analyzed as a whole to determine which of these properties are relevant to the phenotype of interest. Such an analysis typically strives to generate hypotheses on the mechanistic processes for the phenotype of interest, which should be further validated in replication studies or functionally interrogated in laboratory experiments. A number of GSA methods have been developed for gene expression data, and have led to novel biological hypotheses about important clinical conditions. These methods have also suggested new avenues for therapeutic intervention on the basis of the unexpected involvement of biological functions and pathways in a variety of disease processes. [13, 16, 17, 31, 34, 35, 41, 42, 44, 47].

While there has been well over a decade of development and application of gene set analysis methods, there are few formal evaluations and comparisons of the commonly used tools and algorithms. The few comparative papers published so far offer a mostly theoretical evaluation of GSA methods and statistical evaluations are rare and limited in scale. [21, 22, 30, 39, 45] Additionally, the vast majority of comparative studies have used benchmark data, as opposed to simulated data for comparison. For example, Tarca et al. (2013) compared sixteen methods (including methods from the over representation analysis and functional class scoring categories [30]) using 42 datasets retrieved from the Gene Expression Omnibus (GEO) for different disease phenotypes. The authors utilized the Kyoto Encyclopedia of Genes and Genomes (KEGG) [29] and Metacore® Disease Biomarker Networks (https://portal.genego.com) knowledge bases. The authors find that the Gene Set Enrichment Analysis (GSEA) [49] and sigPathway [51] methods have inflated false positive rates along with PLAGE (Pathway Level Analysis of Gene Expression) (Tomfohr et al., 2005), GLOBALTEST (Goeman et al., 2004), PADOG (Pathway Analysis with Downweighting of Overlapping Genes) (Tarca et al., 2012) and MR-GSE (Mean-Rank Gene Set Enrichment) (Michaud et al., 2008) as the best overall methods. Additional studies have proposed frameworks for conducting GSA based on method evaluations from real gene expression datasets, as well as synthetic datasets, where the signal and correlation structure have been simulated [2]. Varemo et al. [52] performed a wide-ranging assessment of multiple approaches in GSA and identified important differences between commonly used methods. Although important differences have been highlighted by all these studies, the use of real datasets without the alteration of signal cannot provide an accurate estimation of statistical power or sensitivity, since it is not known which gene sets are indeed enriched. Simulation experiments are a crucial component of methods evaluation and comparison, as bias, variance, and power properties can only be assessed in simulations with known parameters. However, the few simulation experiments that have been performed have utilized artificially constructed gene sets that may not be reflective of real biological mechanisms [2, 19, 39, 53]. For example, genes are not independent and have complex correlation structures. The data correlation structure in these previously conducted studies was purely synthetic as opposed to incorporating correlations resulting from true biological mechanisms.

Although comparing GSA methods using real gene expression datasets ensures that the comparisons are being performed on biologically relevant data, it is impossible to know what the true underlying signal really is. This limitation prevents an in depth comparison of the results from each method, as no true assessment of the sensitivity or specificity can be used to determine which method performs best. On the other hand, purely synthetic datasets provide the advantage of tuning a variety of conditions for systematic methods comparisons. However, biological systems are highly complex and we cannot be sure that purely synthetic datasets provide accurate representations of the biology these methods will encounter in practice. For these reasons, a better approach is to maintain the underlying correlation structure within genes, while varying the amount of signal for a given gene set. This also provides the advantage of being able to assess each GSA method for detecting off-target gene sets. Without sufficient understanding of the statistical properties of GSA, we risk drawing erroneous conclusions (either false positives or false negatives), which may subsequently lead to unnecessary investments in functional follow-up studies and/or missed oppurtunities.

In this study, we evaluate and compare the statistical properties of some of the most commonly used GSA methods and corresponding software packages, including Gene Set Enrichment Analysis (GSEA) [49], Significance Analysis of Function and Expression (SAFE) [6], sigPathway [51], and Correlation Adjusted MEan RAnk (CAMERA) [53]. Not only do these methods represent many of the commonly used GSA approaches, but they each take a unique approach to performing GSA, providing a diverse set of conditions to test the proposed simulation framework. We take an “end-user” focused approach to the comparison, beginning with default/author recommended parameter settings for each of the methods. We generate a wide range of simulated datasets, using real data from Gene Expression Omnibus (GEO) [3] to provide realistic genome-wide expression profiles for the simulations. We then simulate a range of effect sizes for a gene set chosen from a commonly used knowledge base, varying a number of parameters in the experiment. Our results clearly demonstrate the overall and relative performance of the methods. For several methods, the results highlight concerns with the default parameter settings, therefore alternative implementations were performed and the results compared. These results mark the first benchmark of GSA approaches using data simulated based on real gene expression data along with signal relevant to known gene sets. The results are intended to provide important guidance to end-users of GSA approaches.

## Methods

Key elements of GSA include the knowledge base (known gene sets that will be tested against), the type of hypothesis being tested, the statistical method used, and the method of controlling false positive error rates. A detailed review of the broad classes of methods, and discussions of their statistical properties be found in Khatri et al. [30]. GSA methods can be classified into three loosely defined generations: over-representation analysis (ORA), functional class scoring (FCS) and pathway-topology (PT) methods. ORA methods typically test whether elements in the datasets are over-represented in a given pathway from a knowledge base. FCS methods commonly strive to detect changes to elements in the dataset that cause alterations in the given pathway from the knowledge base. PT methods incorporate known or estimated structures of the biological network to account for correlations among genes.

A number of knowledge base resources have been established by government, academic and private entities. These include gene ontology (GO) [1], Kyoto Encyclopedia of Genes and Genomes (KEGG) [29], MetaCyc [11], Metacore® Disease Biomarker Networks (https://portal.genego.com), Ingenuity Pathway Analysis (IPA®, QIAGEN Redwood City, https://www.qiagenbioinformatics.com/products/ingenuity-pathway-analysis/) and Molecular Signatures Database (MSigDB) [49]. MSigDB (version 5.0) is used here since it includes information from the other databases (total of 10,295 gene sets) and is widely used. By using biologically defined sets, the simulation results parallel the procedure undertaken by typical end-users. MSigDB contains eight different categories of gene sets: positional (c1, 326 sets), literature curated (c2, 4726 sets), motif (c3, 836 sets), computation (c4, 858 sets), GO (c5, 1454 sets), oncogenic (c6, 189 sets), immunologic (c7, 4872 sets), and hallmark (h, 50 sets). A more detailed description of the MSigDB database can be found in Subramanian et al. [49].

Each GSA method evaluated here relies on gene sets from MSigDB, and then tests for whether the gene sets are significantly associated with the disease or trait of interest. Each method takes a slightly different approach to statistical testing, corresponding to different null hypotheses. Two common null hypotheses across the GSA methods are self-contained and competitive. [21, 22] A self-contained null hypothesis states that genes in the gene set are not more differently expressed than what is expected to randomly occur. A competitive null hypothesis states that the genes in a given gene set are not more differently expressed than the other genes in the dataset. The competitive null hypothesis answers questions regarding the pathways compared to each other, while the self-contained hypothesis answers more general questions regarding the activities of genes within each specific pathway. Typically, competitive tests are more conservative compared to self-contained and both tests are sensitive to the number of gene sets tested and the number of genes in the dataset [30]. Given their comparative nature, care should also be taken in interpreting the results from competitive tests. Therefore, controlling the false positive error rates is a challenging task in either context, and this is further complicated due to the high level of overlap of genes contained in different gene sets. To control the false positive error rate resulting from testing multiple hypotheses, family-wise error rate control, using, e.g. Bonferroni correction [14, 15], False Discovery Rate (FDR) control [7] and resampling [24] or permutation-based methods [18, 23] are used. As mentioned above, we chose the most commonly used GSA methods for our comparison, and used the authors corresponding software packages.

### Gene set enrichment analysis (GSEA)

GSEA ranks all of the genes in the dataset based on differential expression. To test the gene set significance, an enrichment score is defined as the maximum distance from the middle of the ranked list. Thus, the enrichment score indicates whether the genes contained in a gene set are clustered towards the beginning or the end of the ranked list. Both self-contained and competitive hypothesis tests can be conducted with GSEA by altering how randomization is completed for hypothesis testing. For a self-contained hypothesis the phenotype labels are permuted while the genes are permuted for a competitive hypothesis. A total of 1000 permutations are performed to estimate the empirical *p*-values for the gene sets. Details of GSEA can be found in Subramanian et al. [49].

### Significance analysis in function and expression (SAFE)

SAFE is a two-stage method which includes calculating both local and global statistics. This two-stage procedure allows the analysis of a set of genes as opposed to single gene association. The local statistic describes the significance of association with the response for each gene in the dataset. The local statistics implemented by the SAFE Bioconductor package [5], include Student’s t-test, Welch’s t-test, paired t-test, F-statistic from ANOVA, and t-statistic from a linear model. In results reported here, the Student’s t-test is utilized as the local statistic since it is the default setting in the software. The global statistic describes the significance of a competitive hypothesis test for each gene set or pathway. The implemented global statistics includes Wilcoxon rank sum, Fisher’s Exact Test, Pearson’s Chi-squared type statistic and a t-statistic for average difference and are all reported in the results for comparison. Permutation of the class labels was conducted to control for false positive error rate. Details of the SAFE method can be found in Barry et al. [6], while details about the package can be found in Barry et al. [5].

### sigPathway

sigPathway offers both a competitive hypothesis test and a variation of a self-contained test, although only the competitive hypothesis was used here since it is the most commonly used. First, a gene-level statistic for the association of each gene with the phenotype of interest is calculated. Second, a statistic is calculated for each gene set by conducting a weighted sum of the gene-level statistics, normalizing this gene set statistic to account for the size and correlation structure of the gene set, ranking the normalized gene set statistics and determining the significance of the gene set statistics by resampling. The default settings use a t-statistic for the gene-level and the Wilcoxon Rank Sum for the gene set level statistics. Details of the sigPathway methodology can be found in Tian et al. [51], and details about the package can be found in Lai et al. [33].

### Correlation adjusted MEan RAnk (CAMERA)

As with SAFE, CAMERA is a two-stage procedure, with gene level and gene set level statistics. The gene level statistic is based on linear regression, testing whether the coefficient of the gene is zero, which is the same as a *t*-test but more flexible. The gene level statistic includes the least squares estimate of the fold change, t-statistic, moderated t-statistic (using an empirical Bayes posterior estimate of the variance) and a normalized moderated t-statistic. CAMERA adjusts the gene set test statistic for inter-gene correlations. The inter-gene correlation is estimated by the variance inflation factor (VIF) and incorporated into the parametric or rank-based hypothesis test. The VIF is calculated using the correlation structure of the dataset, which can be efficiently estimated by the QR-decomposition of the design matrix (that of the linear model corresponding to the gene set) and the independent residuals from this linear model. A number of gene set tests, including Wilcoxon rank sum, are implemented in the software, and include extensions of a t-test between two gene sets group, each containing the gene wise statistics of the genes that they contain and their inter-gene correlation. More details about the methodology of CAMERA can be found in Wu & Smyth [53], with implementation details in Ritchie et al. [46].

### Flexible algorithm for novel gene set simulation (FANGS)

**Fig. 1:**
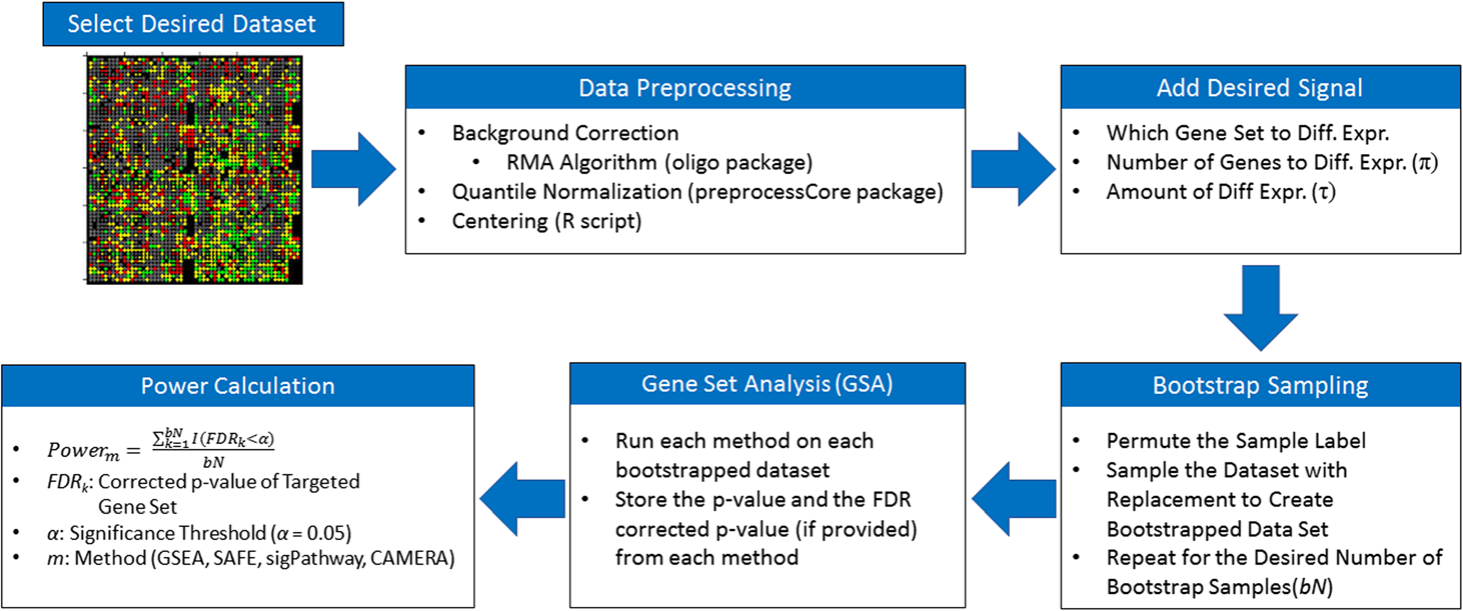
FANGS Flow Chart. A specific dataset is selected as baseline. The raw data is preprocessed as described in the Methods section, which includes RMA and quantile normalization along with centering the data for each gene to have mean zero, to remove the existing signal. Next, a gene set is selected for simulation, and a range of association signals are introduced, with a range of effect sizes, and proportions of the genes in the gene set that are associated with disease status. One hundred bootstrapped data sets are generated for each set of simulation parameters. The simulated data is tested on each method and power to detect the differentially expressed gene set is calculated

FANGS is a semi-synthetic simulation tool, which simulates data based on a user-specified dataset to maintain realistic patterns of variation and correlation. We chose three different datasets to base our simulations on. The first is a prostate cancer dataset from the Gene Expression Omnibus (GEO) database [3] (GSE62872). The dataset contains a total of 424 samples (264 prostate cancer and 160 non-matched normal tissue samples [43]. The other two were samples for a study on ischemic stroke, with 40 samples, (GSE22255; [32]) and 41 samples from normal brain tissue (GSE53890; [37]). Gene expression data for all datasets were collected on the Affymetrix GeneChip Human Gene 1.0 ST Microarray (33,297 probes). All probes that mapped to multiple gene products (as defined by Affymetrix annotation) were removed from the dataset. For the prostate cancer dataset, this resulted in 30,202 probes in the final dataset, of which 19,276 mapped to known genes. For the stroke data, this resulted in 43,494 probes, 36,800 of which mapped to known genes. For the normal tissue dataset, this resulted in 32,866 probes, 20,514 of which mapped to known genes. The simulation process is summarized in Fig. 1. Multiple datasets were chosen to evaluate the consistency of the results across dataset with varying correlation structures, study designs, etc.

The dataset preprocessing included background correction by the RMA algorithm [26, 27] quantile normalization and centering of each probe’s expression at zero to remove the existing signal [20]. RMA adjusts for sources of noise from the microarray including between and within arrays. (Silver et al., 2009; [8, 10, 26, 27]) While there are a number of approaches for normalization, quantile normalization is commonly used because it is robust and the resulting signal is independent of expression technology. The “rma” function within the oligo package [10] in the R programming language was used for background correction. The “normalize.quantiles” function within the “preprocessCore” package [9] in the R programming language was used for the quantile normalization. Centering removes any original signal in the dataset, while preserving the inherent correlation structure amongst genes. Additional file 1: Figure S1 shows a heat map of the correlation structure (calculated by the Pearson correlation coefficient) of the prostate cancer dataset after preprocessing and normalization.

Next, the simulated signal was added to genes in a specific gene set, as defined by the MSigDB database. There are three important parameters in this simulation experiment: 1) gene set selection, 2) proportion of genes within the set that are differentially expressed, and 3) differential expression effect size. For each expression dataset, we selected two gene sets. For the prostate cancer dataset we selected two gene sets that were previously found to be associated with prostate and other cancers [25] (the KRAS down regulated pathway – ‘KRAS.PROSTATE_UP.V1_DN’ in MSigDB and the TGF-β signaling pathway – ‘TGF_BETA_SIGNALING’). The KRAS pathway contains a total of 144 genes, 127 of which are included in the prostate cancer dataset. The TGF-β signaling pathway contains a total of 54 genes, all of which are included in the prostate cancer dataset.

For the ischemic stroke and normal brain tissue datasets two random pathways were selected. A complete list of gene sets in each category in MSigDB was generated, and a random number generator was used to pick the pathway for simulation. The ischemic stroke data targeted the “MORF_ANP32B” from c4 (genes in the neighborhood of ANP32B) and the “MARTORIATI_MDM4_TARGETS_NEUROEPITHELIUM_UP” from c2 (genes which are up-regulated in apoptotic tissues after MDM4 has been knocked out) pathways. The MORF_ANP32B and MARTORIATI_MDM4_TARGETS_NEUROEPITHELIUM_UP pathways contain 197 and 176 genes, 192 and 159 of which are contained in the ischemic stroke dataset, respectively.

The normal dataset targeted “GCM_FANCC” from c4 (genes in the neighborhood of FANCC) and “RAMASWAMY_METASTASIS_DN” from the c2 (genes which are down-regulated in metastatic as opposed to primary solid tumors) pathways. The GCM_FANCC and RAMASWAMY_METASTASIS_DN pathways contains 124 and 61 genes, 119 and 59 of which are included in the normal brain tissue dataset.

By selecting gene sets of different sizes, the impact of the number of genes in a gene set can be evaluated. We also chose gene sets from different categories of the knowledge base to evaluate if the result patterns are consistent across gene set categories. The proportion of genes differentially expressed (π) in the simulations defines the proportion of the genes randomly selected in the gene set to be differentially expressed (as it would not be expected that all the genes in a gene set would be differentially expressed). This proportion ranged from 0.05 to 1.0 in this experiment. A range of signal was then introduced into the data, by shifting the gene expression in the case group according to a range of values (τ). The magnitude (or effect size) (τ) of differential expression given by *τ* ∗ *sd*(*expr*), defines the magnitude of alteration of the expression of each probe.

To assess the power of each of the gene set analysis methods, 100 bootstrapped samples from the differentially expressed datasets are created, keeping the case-control balance from the original study in the case of the prostate cancer data or a balanced design in the other datasets (264, 20, and 21 cases along with 160, 20, and 20 controls in the prostate cancer, ischemic stroke, and normal brain datasets, respectively). Power in all simulations was calculated as the proportion of bootstrapped datasets where the differentially expressed gene set displays a significant (at a threshold of 0.05) gene set level statistic.

Three null simulations were conducted to assess the false positive rate for each of the GSA methods tested here: 1) We simulated a null condition by permuting the class labels without changing the expression level (τ), breaking the association between the expression profile and the response. 2) We sampled the expression for each probe from an independent identically distributed (iid) standard normal distribution and randomly assigned sample labels, breaking the association between the expression profile and the response, as well as breaking the correlation structure between the genes. 3) We normalized and centered the data with τ = 0, thus adding no signal to the data. The power under the null conditions was calculated as the proportion of pathways in the null simulations with a *p*-value below 0.05.

### Implementation

The software to create simulated datasets was implemented in the R statistical programming language (https://www.r-project.org/) version 3.2.2. Version 1.0 of FANGS is available open-sourced online at https://github.com/rmathur87/FANGS. The algorithm is flexible to incorporate user defined data along with other knowledge base configurations. The parameters of the simulation can be easily altered to test different values, for example sample size or different sampling schemes.

In all simulations presented here, one hundred bootstrapped replicates were generated using each combination of τ, and π for the two selected gene sets. The τ values tested included 0.25, 0.5, 1, 2, and 10. The π values tested included 0.05, 0.1, 0.25, 0.5, and 1. The GSA methods were tested using the implementations provided by the original authors with the recommended default parameters (Table 1), and alternate parameters as appropriate (Table 2). SAFE [5], sigPathway [33] and CAMERA [46] were run using Bioconductor. GSEA (Java) was downloaded from (http://software.broadinstitute.org/gsea/). The alternative parameters were utilized with SAFE as suggested in Barry et al. [4]. All computation times were estimated on Dell’s PowerEdge R620 rack servers which includes two Intel Xeon processors for a total of 16 cores and 128 GB of RAM.

**Table 1:** List of default parameters

Method	Default Parameters
GSEA	Data File: Inputted txt file Response File: Inputted txt file Knowledge base: mSigDB gmx file Num. Permutations: 1000 Permutation Type: Gene or Phenotype Scoring Scheme: Weighted Ranking Metric: Signal To Noise
SAFE	Data Matrix (X.mat): Inputted rda file Response Vector (y.vec): Inputted rda file Gene Category Assignments (C.mat): Created from gmt file Method: Permutation Min Category Size: 2 Max Category Size: Inf by.gene: FALSE Local Statistic: Student T-Test (“t.Student”) Global Statistic: Wilcoxon Rank Sum (“Wilcoxon”) Args.global: list(one.sided = F) Num. of Permutations (‘Pi.mat’): 1000 Multiple Testing Correction (‘error’): FDR.BH
sigPathway	Data File: Inputted rda file Knowledge base: mSigDB gmx file Min gene set size: 1 Max gene set size: 10,000 allPathways: TRUE Number of Pathways: length(mSigDB gmx file)
CAMERA	Data File: Inputted rda file Response Inputted rda file Knowledge base: mSigDB gmx file

**Table 2:** List of alternative parameters

Method	Alternative Parameters
GSEA	Calculating the FDR q-values based on the reported p-values as opposed to the q-values reported, which are based on the median.
SAFE	Method: Bootstrap (‘Bootstrap.q’) Global Statistic: aveDiff, FET, Pearson Num. of Permutations (Pi.Mat): 10,000
sigPathway	Not applicable
CAMERA	Not applicable

To explore whether 100 bootstrap samples is enough to accurately estimate power, a subset of simulations were repeated using 1000 bootstraps, and the results were compared to those run using 100 bootstraps. There were no significant differences in the power estimate (α > 0.05) for any of the GSA methods (results not shown).

## Results

A complete reporting of results for all three datasets is included in Additional file 1, but because the trends are extremely similar and the overall conclusions identical, for simplicity we focus on the prostate cancer data results here in the main text.

**Fig. 2:**
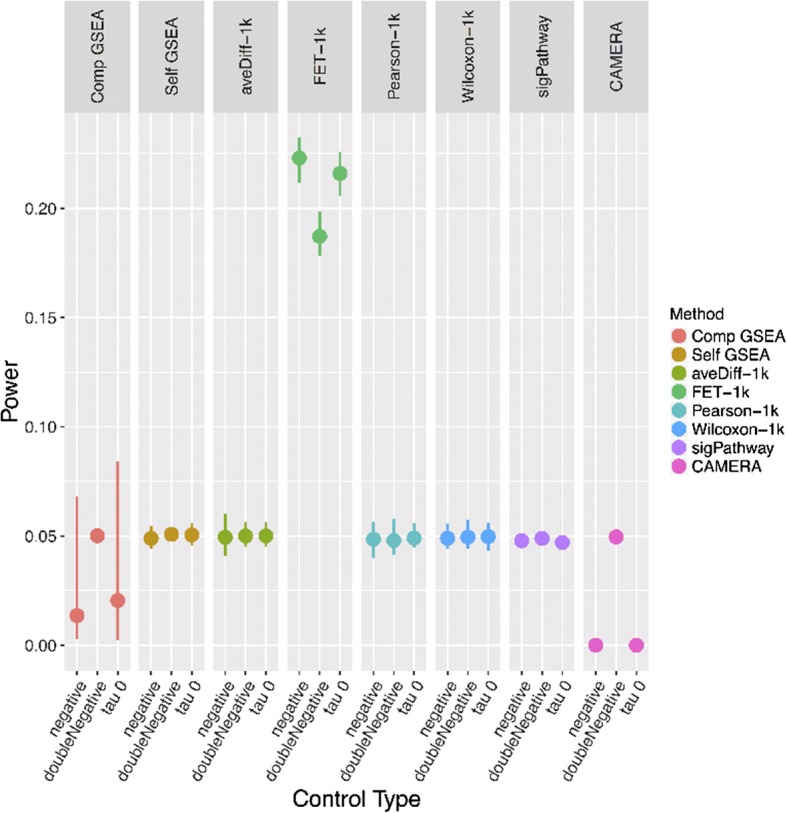
Power under null simulation. Statistical power as detected by null simulation data for all GSA methods tested. The mean power (for 100 bootstrap datasets) for the three different null simulations is plotted along with its 95% confidence interval for the mean of each method

To fairly evaluate power, first the overall Type I error rate of each method needs to be assessed. Figure 2 displays the results for the three different null simulations across each of the methods (all tests implemented SAFE global statistics with default settings, alternative settings are shown in Additional file 1: Figure S2). The median power across all 100 replicates from all gene sets within MSigDB is displayed (along with first and third quantiles). All methods except for the FET global statistic in SAFE have median power around 0.05 (raw values shown in Additional file 1: Table S1), demonstrating that generally false positive rates are well controlled. This is in opposition to the findings of Tarca et al. (2013) who found that sigPathway and GSEA had inflated false positive rates.

**Fig. 3:**
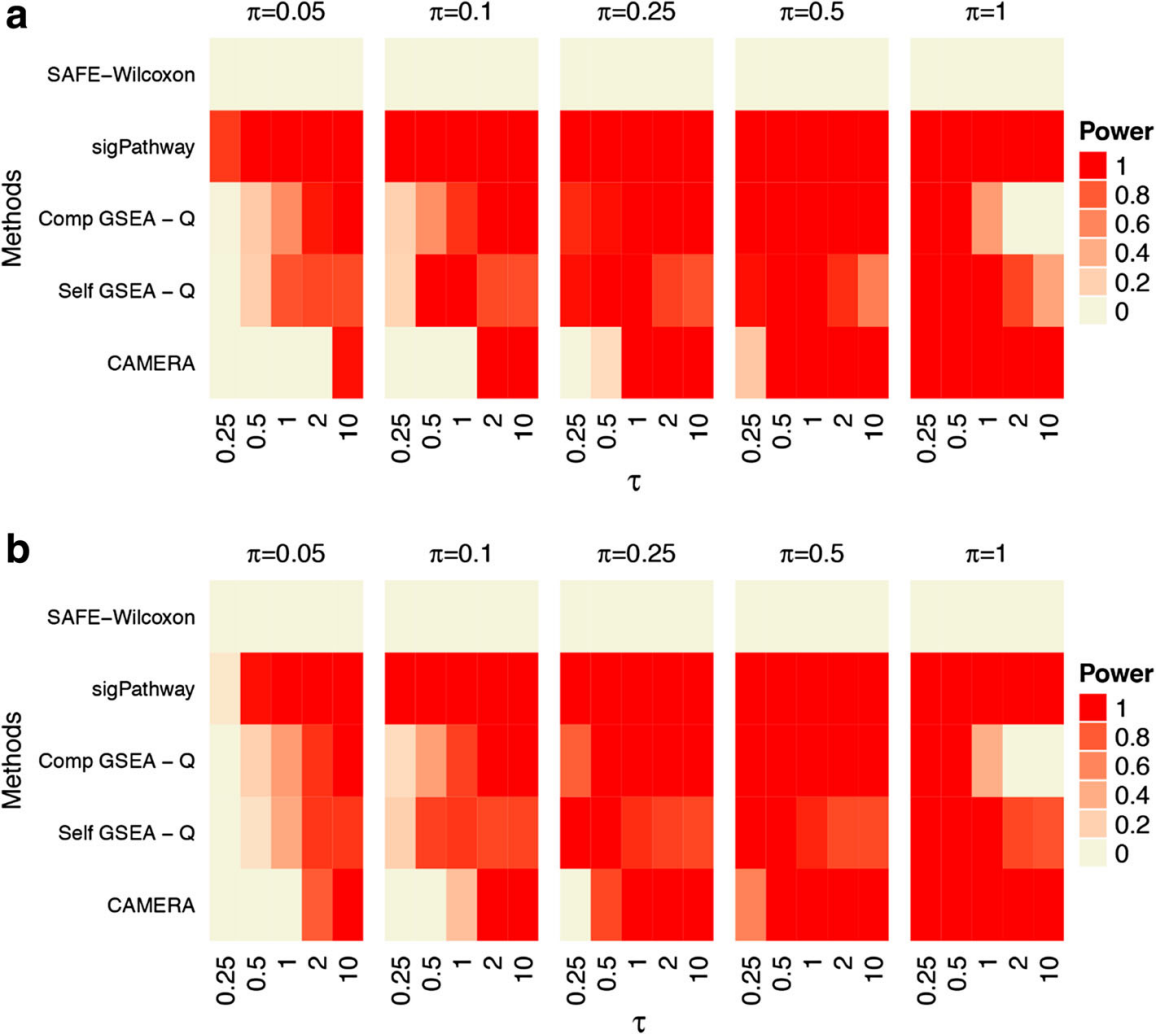
Power for Default Settings. Statistical power for all GSA methods tested under the default settings. All power values are shown at a significance value of 0.05. **a** The KRAS.PROSTATE_UP.V1_DN pathway has been differentially expressed. **b** The TGF-β SIGNALING pathway has been differentially expressed

Power results across the range of simulations using the default parameter settings are shown in Fig. 3 for the prostate cancer dataset. The results for the other two dataset are shown in Additional file 1: Figures S3 through S5. The raw values for the prostate cancer dataset are shown in Additional file 1: Tables S2 and S3 and are available upon request for the other datasets. Results for the KRAS pathway simulations are shown in Panel (a) and for the TGF-β signaling pathway in Panel (b). Overall, trends are as expected. Power is higher for the KRAS pathway than the TGF-β pathway. This is expected given its larger size, and the fact that a fixed proportion of genes in each pathway is set to be differentially expressed in our simulations. Also, as the proportion of differentially expressed genes in the gene set increases, power for each of the methods increased. Generally, as the effect size increases, power increases (though an exception is discussed below). These same trends are observed from the MORG_ANP32B, MDM4, GCM_FANCC, and METASTASIS pathways in Additional file 1: Figures S3 through S6.

The relative performance of the methods is also evident, with a few notable trends. The Wilcoxon global statistic for SAFE discovers no signal at any tested levels of τ, supporting previous reports that SAFE is a conservative approach [30]. CAMERA has power to detect larger effect sizes (τ = 1,2,10) although its power diminishes for smaller effect sizes. sigPathway displays high power to detect a wide range of signal strengths. Generally, this is also true for GSEA, though this trend reversed for very strong signals (π = 1; τ = 2,10).

**Fig. 4:**
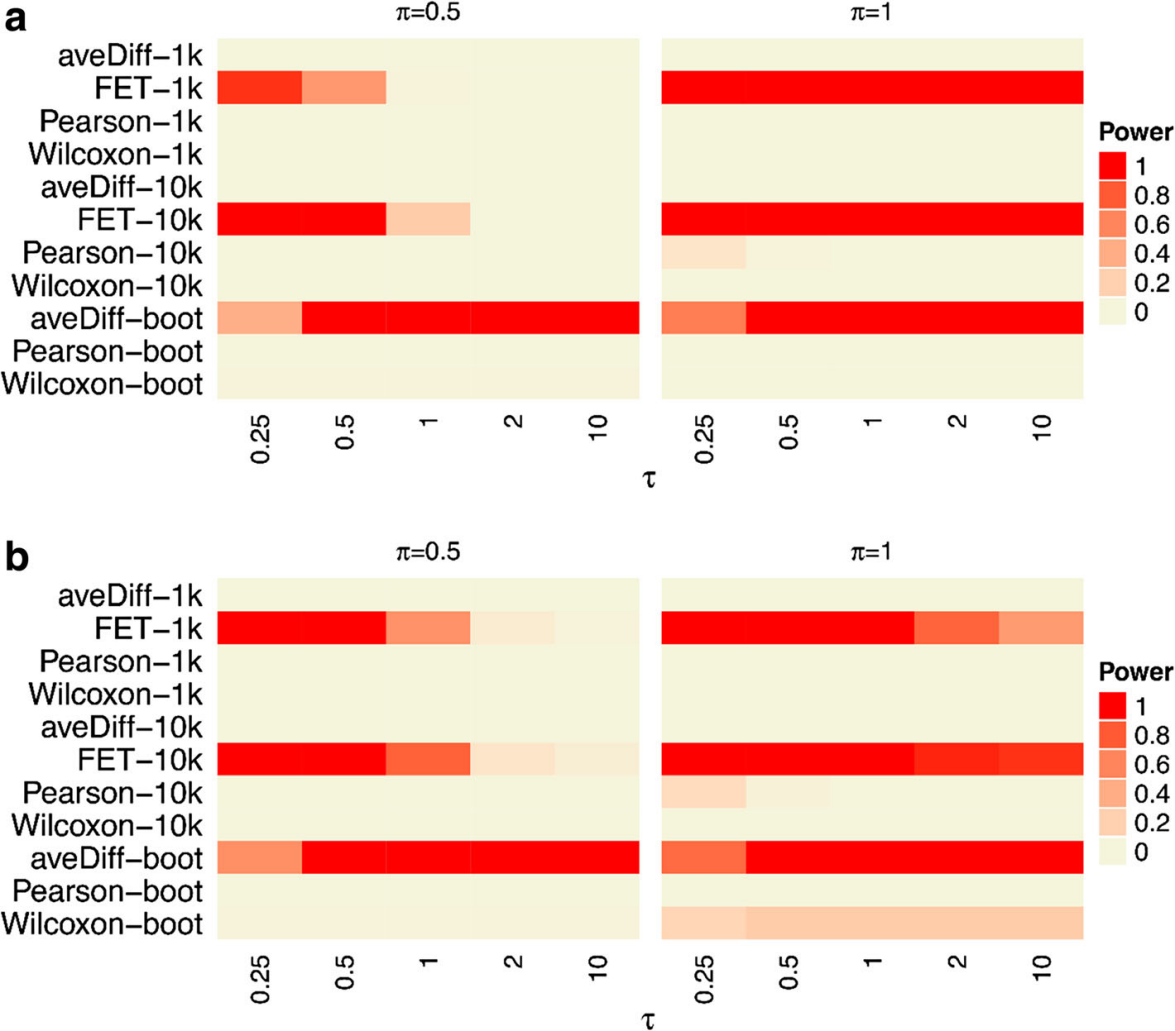
Power for SAFE alternative settings. Statistical power for results with alternative settings alongside default settings for SAFE. The alternative settings tested include 10,000 permutations (‘-10 k’ labels) and bootstrap sampling (‘-boot’ labels). The default setting is with 1000 permutations (‘-1 k’ labels). All power values are shown as a significance value of 0.05. **a** The KRAS.PROSTATE_UP.V1_DN pathway has been differentially expressed. **b** The TGF-β SIGNALING pathway has been differentially expressed

Simulation with the default settings for SAFE produced extremely low power (Fig. 4), prompting the exploration into other parameter settings, including increasing the number of permutations (increased *p*-value resolution), conducting other global statistical tests to assess sensitivity and determine if bootstrap sampling improves power for large signals. [4] The results with alternative and default parameters using SAFE for the KRAS down regulated and TGF-β signaling pathways are shown in Fig. 4. For large effect sizes the bootstrap sampling proves to have higher power compared to permutation testing (even with a larger number of permutations), which supports previous work with bootstrap sampling [4]. The power does decrease significantly for lower signals (π = 0.5), therefore the signal must be relatively strong for SAFE to detect it.

**Fig. 5:**
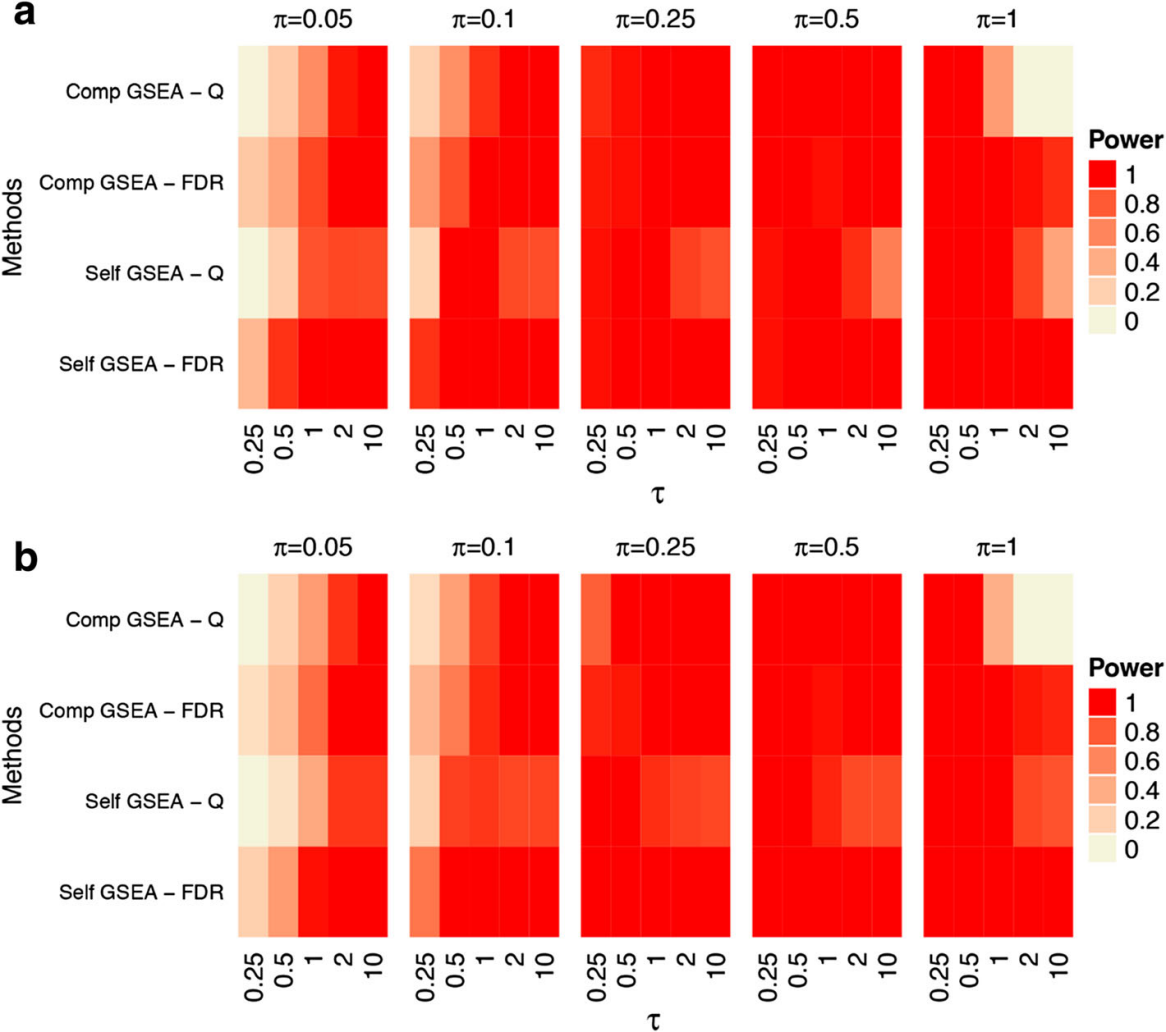
Power for GSEA alternative settings. Statistical power for results with alternative settings alongside default settings for GSEA. The alternative setting consists of calculating the FDR q-values based on the extremes as opposed to the median reported in GSEA. All power values are shown as a significance value of 0.05. **a** The KRAS.PROSTATE_UP.V1_DN pathway has been differentially expressed. **b** The TGF-β SIGNALING pathway has been differentially expressed

Further examination of the results from GSEA highlighted an unusual property of the false discovery rate (FDR) q-value as calculated by GSEA. GSEA reports q-values based on the median of the p-value distribution, as opposed to the standard approach of using the extreme values [7]. To evaluate whether the unexpected results were due to this method of q-value calculation, we repeated the GSEA analysis with a traditional q-value implementation. [7] We calculated FDR q-values based on the reported permutation-based *p*-values via the stats package (“p.adjust” function) in the R programming language. Differences in the powers between the two implementations of FDR are shown in Fig. 5, and demonstrate that the unusual behavior is corrected with the more commonly used FDR implementation.

**Fig. 6:**
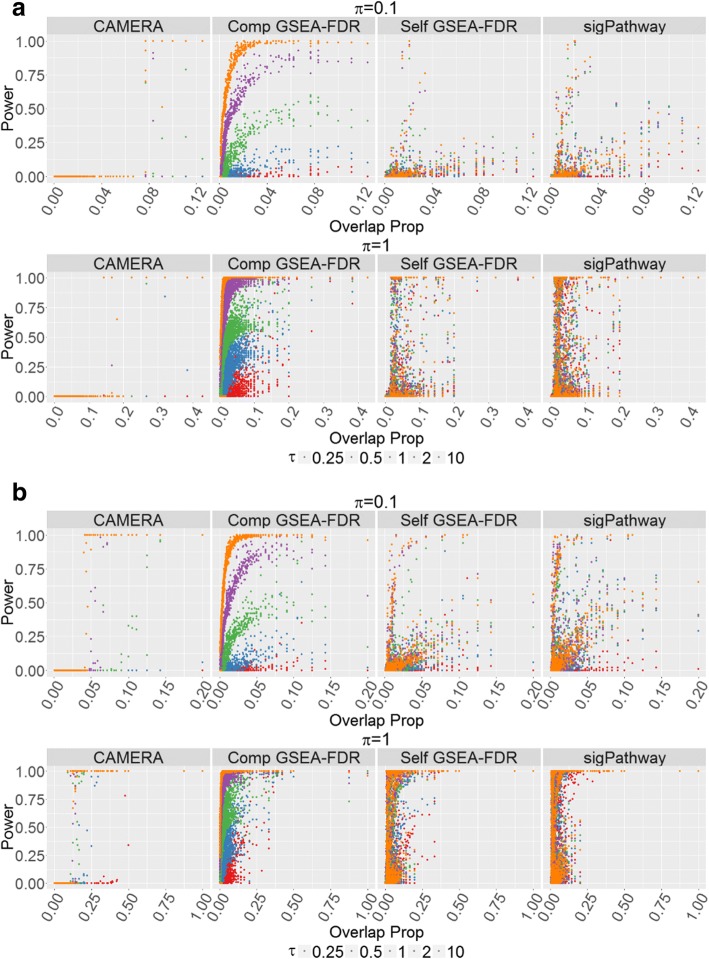
Power of Overlap Pathways. Statistical power for results with recommended methods (CAMERA, GSEA with user defined FDR q-value and sigPathway) for those pathways with genes overlapping the targeted pathway and denoted by the overlap proportion. Only proportion of differential expression parameter (π) values of 0.1 and 1 are shown here, while the others are displayed in Additional file 1: Figures S7 and S8. All power values are shown as a significance value of 0.05. **a** The KRAS.PROSTATE_UP.V1_DN pathway has been differentially expressed. **b** The TGF-β SIGNALING pathway has been differentially expressed

Genes are not always unique to a given gene set or pathway, and are often included in multiple gene sets, posing a challenge to assessing GSA methods. To examine how secondary gene sets not targeted in the simulation are impacted, the powers for gene sets overlapping with target gene sets are shown in Fig. 6 for the most powerful methods: sigPathway, GSEA and CAMERA for large (π = 1) and small (π = 0.1) proportion of differentially expressed genes. The sensitivity of these methods to detect these secondary gene sets is not surprising since some of these gene sets contain differentially expressed genes. The competitive GSEA is the most sensitive to these secondary gene sets, while sigPathway and self-contained GSEA detect signal only for very large effect sizes. Lastly, CAMERA has the lowest relative power to detect these secondary gene sets.

In addition to statistical considerations, computational run-times can be an important consideration for end-users. Table 3 lists the computational times for each of the software packages assessed here.

**Table 3:** Average run time for each GSA method

Method	Ave Run Time
SAFE	Default Global Statistics • aveDiff: 18 mins • Pearson: 16 mins • FET: 17 mins • Wilcoxon: 17 mins 10,000 Permutations: 149 mins 10,000 Bootstraps: 134 mins
sigPathway	4:08 mins
GSEA	Competitive: 39:58 mins Self-Contained: 34:28 mins
CAMERA	00:15 mins

## Discussion

GSA has become an important tool in gene expression analysis, and GSA approaches tied to knowledge bases such as MSigDB, are some of the most popular approaches [30, 45]. While there have been a number of theoretical discussions of the differences amongst the methods, and benchmark datasets have been used to demonstrate the relative performance of methods, there have been few simulation experiments that compare these popular methods. Simulation experiments provide important guidance in choosing methods, and choosing parameter settings for such methods.

In the current study we took a very practical approach to the methods comparison. We used the implementations and recommended parameter setting for several of the most commonly used GSA methods, and used real data for semi-synthetic simulations. This is an advantageous strategy compared to the use of benchmark datasets since false positive rates and power can be accurately estimated, providing a more rigorous approach to comparing methods. There are also advantages to the semi-synthetic simulations implemented in our FANGS approach compared to the purely synthetic simulations that have been performed before. The use of real data in semi-synthetic simulations preserves the correlation structure of genes across the genome as well as other complexities of the data (relative distributions, real levels of noise, technical biases, etc).

The default parameters for SAFE failed to detect any of the effect sizes tested (Fig. 3), although the bootstrap sampling improved performance for larger effect sizes (Fig. 4). The default parameters of GSEA (both self-contained and competitive) produced surprisingly low power for very large effect sizes, although its relatively high power was observed at lower effect sizes (Fig. 3). This result seems to be due to the calculation of q-value based on the median in the distributed software, as opposed to the more commonly used approach of basing the calculations on the extreme values of the *p*-values. [7] Implementing GSEA with the FDR q-value, as developed by Benjaminini and Hochberg, resulted in more predictable power behavior and overall improved power (Fig. 5). We believe the improvement occurs because the median p-value used in the q-value calculation by GSEA is insensitive to changes in p-values at the extreme of the distribution (i.e. smaller p-values). Subsequently, the increased signal is dampened as more significant p-values are observed. Assessing the p-values at the extremes of the distribution, as is performed in the FDR approach developed by Benjamini and Hochberg, is more sensitive to detecting changes where increasing significant p-values occur at the extreme of the distribution. CAMERA performed well with larger effect sizes, but demonstrated relatively poor performance with small to intermediate effect sizes (Fig. 3). sigPathway had the highest power of all the methods across the full range of tested effect sizes (Fig. 3). The competitive GSEA and sigPathway methods were more sensitive than other methods, and also detected signal in other gene sets with genes that overlapped with the target gene set (Fig. 6). Differences were observed across all methods in their power to detect the TGF-β Signaling pathway and the KRAS pathway due to the different pathway sizes. Importantly, all the methods except for the SAFE-FET implementation controlled the Type I error in our experiments.

Based on these results, we can now form practical recommendations for end users. Overall, the most powerful method was sigPathway; however, GSEA with the user-calculated q-values has very similar performance and detects secondary signals from different gene sets. Therefore, GSEA displays better performance for identifying the specific signal along with other correlated and related gene sets, which can be desirable for those seeking hypothesis generation. However, this can also complicate the interpretation of “enriched” pathways identified using GSEA, as it is unclear whether the enrichment is due to signal in the pathway, or secondary enrichment due to correlation or overlap. On the other hand, sigPathway is more powerful in detecting smaller effect sizes in the dataset. Furthermore, sigPathway has a relatively shorter computational runtime of about five minutes compared to about forty-five minutes for GSEA, about fifteen minutes for 1000 permutations and over two hours for 10,000 permutations or bootstrap sampling for SAFE (Table 3). CAMERA is the most efficient algorithm with a run time of 16 s for a single dataset. Based on the comparison of the two different q-value implementations within the GSEA method and using the classical FDR controlling approaches, we recommend that users implement their own FDR correction, and not use the q-values calculated within the software package.

While we tried to simulate a reasonable range of simulation parameters, there are a number of factors that were not included in the current study. Future studies should focus on the effect of sample size on the resulting statistical power. Additionally, other real datasets should be used as the basis of the simulations to evaluate the impact of various correlation structures, different sample sizes, missing data, preprocessing choices (e.g. normalization), limits of detection, and class misspecification should also be considered. The results were remarkably similar across the three datasets used here, but this is still a limited set of datasets. Importantly, future studies should extend the types of data simulated and analyzed. For instance, while microarrays have the longest history of GSA, RNA-Seq data has different structures and variance properties that have prompted the development of different GSA methods [48]. This necessitates the evaluation of RNA-sequencing data, and other platforms, such as proteomics and metabolomics, to be assessed in a similar framework.

There are also limitations to the semi-synthetic approach we implemented in general, especially compared to previous purely synthetic experiments. In our implementation, while the correlation structure across the genome is preserved, the differentially expressed genes in the simulated gene sets are not correlated. Previous simulations have demonstrated the correlation amongst genes in the gene set to be evaluated is an important factor in the power of each methods, with general and very consistent trends that as correlation increases, the power of both self contained and competitive methods decreases [39]. This is a general trend across all methods, and typically does not change the relative performance. Incorporating additional simulation options like this will be an important future direction for the continued development of FANGS. We simulated a limited set of simulations in for the TGF-β signaling pathway data with (π = 1; τ = 5) adding correlation to the gene expression values in the gene set (*r* = .2 and .4) and the results followed the expected trend, where power decreased as correlation increased, but the relative performance of the methods was the same (results not shown).

Additionally, while we tried to implement some of the most commonly used GSA methods, this is an active area of development. Additional methods could be considered for comparison as permitted. In particular, a systematic evaluation of pathway topology methods [30], such as DEgraph [28], NetGSA [38], SPIA [50] would be informative. There are also a number of commercial software packages that are available, including Ingenuity Pathway Analysis (IPA®, QIAGEN Redwood City, https://www.qiagenbioinformatics.com/products/ingenuity-pathway-analysis/) that are popular, but licensing agreements prevent benchmarking studies from being conducted.

Finally, we have only used a single knowledge base for the methods comparison – MSigDB. We used the literature curated (c2), motif (c3), computation (c4), GO terms (c5), oncogenic (c6), immunologic (c7), and hallmark (h) categories within MSigDB. There are a range of possible knowledgebases available, many specifically geared for new technologies (e.g. metabolomics) or for specific genetic systems (e.g. plants). Comparing the performance of the different approaches with a range of knowledge bases, and with limited categories within the knowledge bases will be an important next step.

There is one other opportunity with the semi-synthetic simulations that should be noted. The FANGS code could also be used to perform power calculations based on pilot data. As any real data can be uploaded, simulated signal could be added in conjunction with resampling to perform simulation-based power calculations for study design. Adding this option to FANGS will be another important future direction.

## Conclusions

We have developed software to create semi-synthetic simulations based on real data to compare the performance of some of the most popular pathway analysis methods. The most powerful methods are sigPathway and GSEA, who have similar performance and detect secondary signals based on pathway overlap. By taking an applied approach to methods comparison, valuable information is gained about the power of each GSA method. With such information more confidence is carried into any functional follow-up studies.

## Additional file

Additional file 1: **Figure S1.** Correlation Structure of Prostate Cancer Dataset. Each row and column refers to genes in the dataset, and the value plotted in the heat map is the Pearson correlation coefficient between the expressions of those genes. The density of correlation values is shown in the top left corner. **Figure S2.** Power of SAFE GSA Method on Negative Controls. Statistical power as detected by negative control data for the alternative parameters for the SAFE GSA method. The mean power (for 100 bootstrap datasets) of the three negative controls is plotted along with its 95% confidence interval for the mean for each method. **Figure S3.** Power for Default Settings from Ischemic Stroke Dataset with the MDM4 pathway targeted. Statistical power for all GSA methods tested under the default settings (including computed GSEA FDR values) from the ischemic stroke dataset with the MARTORIATI_MDM4_TARGETS_NEUROEPITHELIUM_UP pathway targeted for differential expression. All power values are shown as a significance value of 0.05. **Figure S4.** Power for Default Settings from Ischemic Stroke Dataset with the MORF_ANP32B pathway targeted. Statistical power for all GSA methods tested under the default settings (including computed GSEA FDR values) from the ischemic stroke dataset with the MORF_ANP32B pathway targeted for differential expression. All power values are shown as a significance value of 0.05. **Figure S5.** Power for Default Settings from Normal Brain Tissue Dataset with the GCM_FANCC pathway targeted. Statistical power for all GSA methods tested under the default settings (including computed GSEA FDR values) from the normal brain tissue dataset with the GCM_FANCC pathway targeted for differential expression. All power values are shown as a significance value of 0.05. **Figure S6.** Power for Default Settings from Normal Brain Tissue Dataset with the metastasis pathway targeted. Statistical power for all GSA methods tested under the default settings (including computed GSEA FDR values) from the normal brain tissue dataset with the RAMASWANY_METASTASIS_DN pathway targeted for differential expression. All power values are shown as a significance value of 0.05. **Table S1.** Statistical false positive rates for the three controls, negative, double negative and no signal (tau 0). All values are shown at a significance value of 0.05. **Table S2.** Statistical power values for simulation results (for both pathways and all pairs of π and τ tested) for all methods along with GSEA alternative settings. All power values are shown at a significance value of 0.05. **Table S3.** Statistical power values for simulation results (for both pathways and all pairs of π and τ tested) with alternative settings for SAFE. All power values are shown at a significance value of 0.05. **Figure S7.** Power of Overlap Pathways for all effect sizes for KRAS Pathway. Statistical power for results with recommended methods for overlap pathways for experiments with the KRAS pathway differentially expressed. The recommended methods include CAMERA, GSEA with user defined FDR q-value and sigPathway. The x-axis denotes the proportion of the pathway that overlaps with the KRAS pathway. Each point in the graph represents one of these overlapping pathways. All power values are shown as a significance value of 0.05. **Figure S8.** Power of Overlap Pathways for all effect sizes for TGF-β Pathway. Statistical power for results with recommended methods for overlap pathways for experiments with the TGF-β pathway differentially expressed. The recommended methods include CAMERA, GSEA with user defined FDR q-value and sigPathway. The x-axis denotes the proportion of the pathway that overlaps with the TGF-β pathway. Each point in the graph represents one of these overlapping pathways. All power values are shown at a significance value of 0.05. (DOCX 892 kb)
