# Supplementary material for: Gene set analysis methods: a systematic comparison
Source: BioData Min. 2018 May 31;11:8. doi: 10.1186/s13040-018-0166-8 (PMC5984476; doi:10.1186/s13040-018-0166-8)
Supplement: Supplementary file 1 — Figure S1. Correlation Structure of Prostate Cancer Dataset. Each row and column refers to genes in the dataset, and the value plotted in the heat map is the Pearson correlation coefficient between the expressions of those genes. The density of correlation values is shown in the top left corner. Figure S2. Power of SAFE GSA Method on Negative Controls. Statistical power as detected by negative control data for the alternative parameters for the SAFE GSA method. The mean power (for 100 bootstrap datasets) of the three negative controls is plotted along with its 95% confidence interval for the mean for each method. Figure S3. Power for Default Settings from Ischemic Stroke Dataset with the MDM4 pathway targeted. Statistical power for all GSA methods tested under the default settings (including computed GSEA FDR values) from the ischemic stroke dataset with the MARTORIATI_MDM4_TARGETS_NEUROEPITHELIUM_UP pathway targeted for differential expression. All power values are shown as a significance value of 0.05. Figure S4. Power for Default Settings from Ischemic Stroke Dataset with the MORF_ANP32B pathway targeted. Statistical power for all GSA methods tested under the default settings (including computed GSEA FDR values) from the ischemic stroke dataset with the MORF_ANP32B pathway targeted for differential expression. All power values are shown as a significance value of 0.05. Figure S5. Power for Default Settings from Normal Brain Tissue Dataset with the GCM_FANCC pathway targeted. Statistical power for all GSA methods tested under the default settings (including computed GSEA FDR values) from the normal brain tissue dataset with the GCM_FANCC pathway targeted for differential expression. All power values are shown as a significance value of 0.05. Figure S6. Power for Default Settings from Normal Brain Tissue Dataset with the metastasis pathway targeted. Statistical power for all GSA methods tested under the default settings (including computed GSEA FDR values) from the [file 13040_2018_166_MOESM1_ESM.docx]

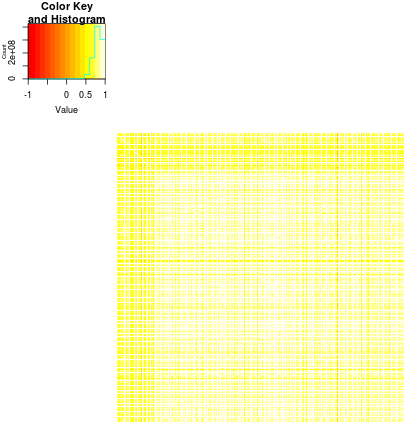


**Supplemental Figure 1: Correlation Structure of Prostate Cancer Dataset.** Each row and column refers to genes in the dataset, and the value plotted in the heat map is the Pearson correlation coefficient between the expressions of those genes. The density of correlation values is shown in the top left corner.


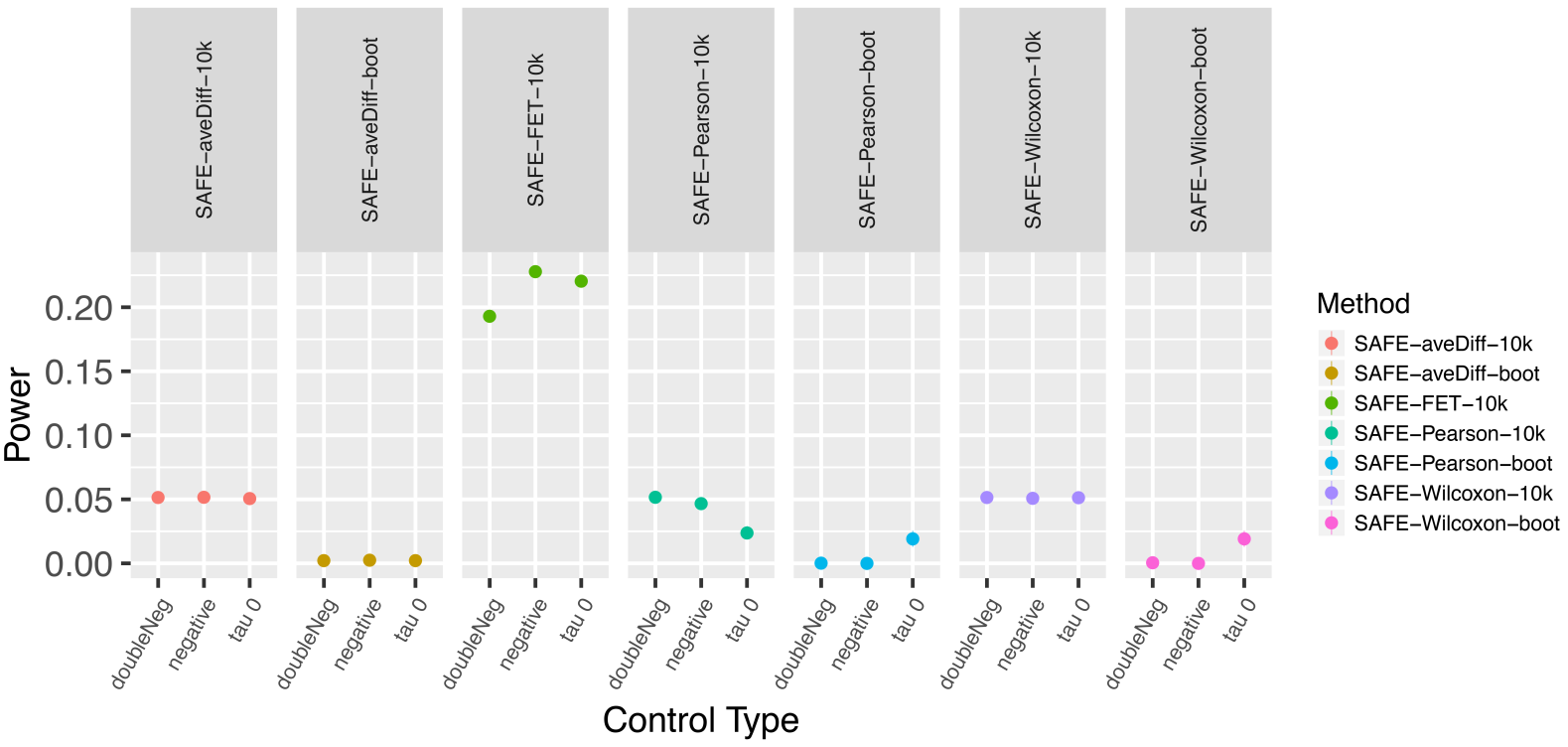


**Supplemental Figure 2: Power of SAFE GSA Method on Negative Controls.** Statistical power as detected by negative control data for the alternative parameters for the SAFE GSA method. The mean power (for 100 bootstrap datasets) of the three negative controls is plotted along with its 95% confidence interval for the mean for each method.


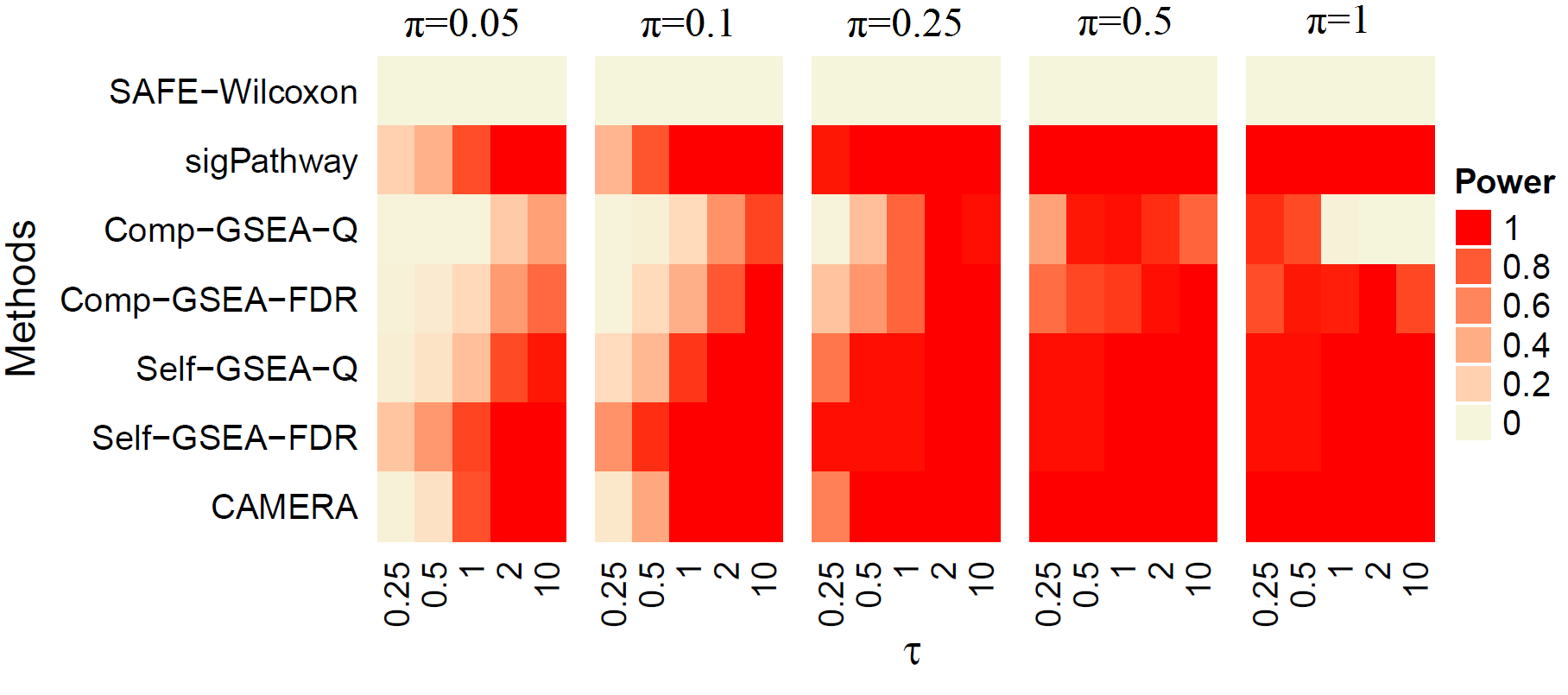


**Supplemental Figure 3:** **Power for Default Settings from Ischemic Stroke Dataset with the MDM4 pathway targeted.** Statistical power for all GSA methods tested under the default settings (including computed GSEA FDR values) from the ischemic stroke dataset with the MARTORIATI_MDM4_TARGETS_NEUROEPITHELIUM_UP pathway targeted for differential expression. All power values are shown as a significance value of 0.05.


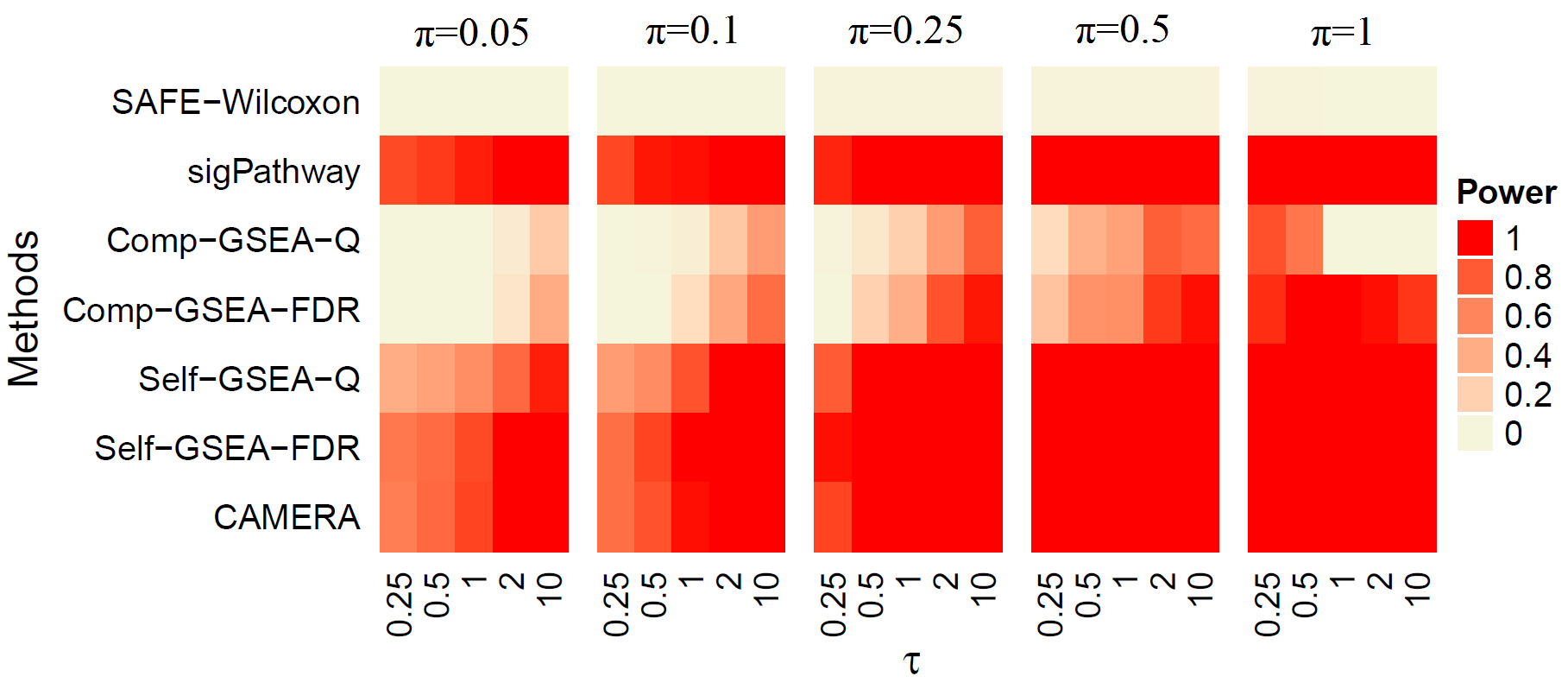


**Supplemental Figure 4:** **Power for Default Settings from Ischemic Stroke Dataset with the MORF_ANP32B pathway targeted.** Statistical power for all GSA methods tested under the default settings (including computed GSEA FDR values) from the ischemic stroke dataset with the MORF_ANP32B pathway targeted for differential expression. All power values are shown as a significance value of 0.05.


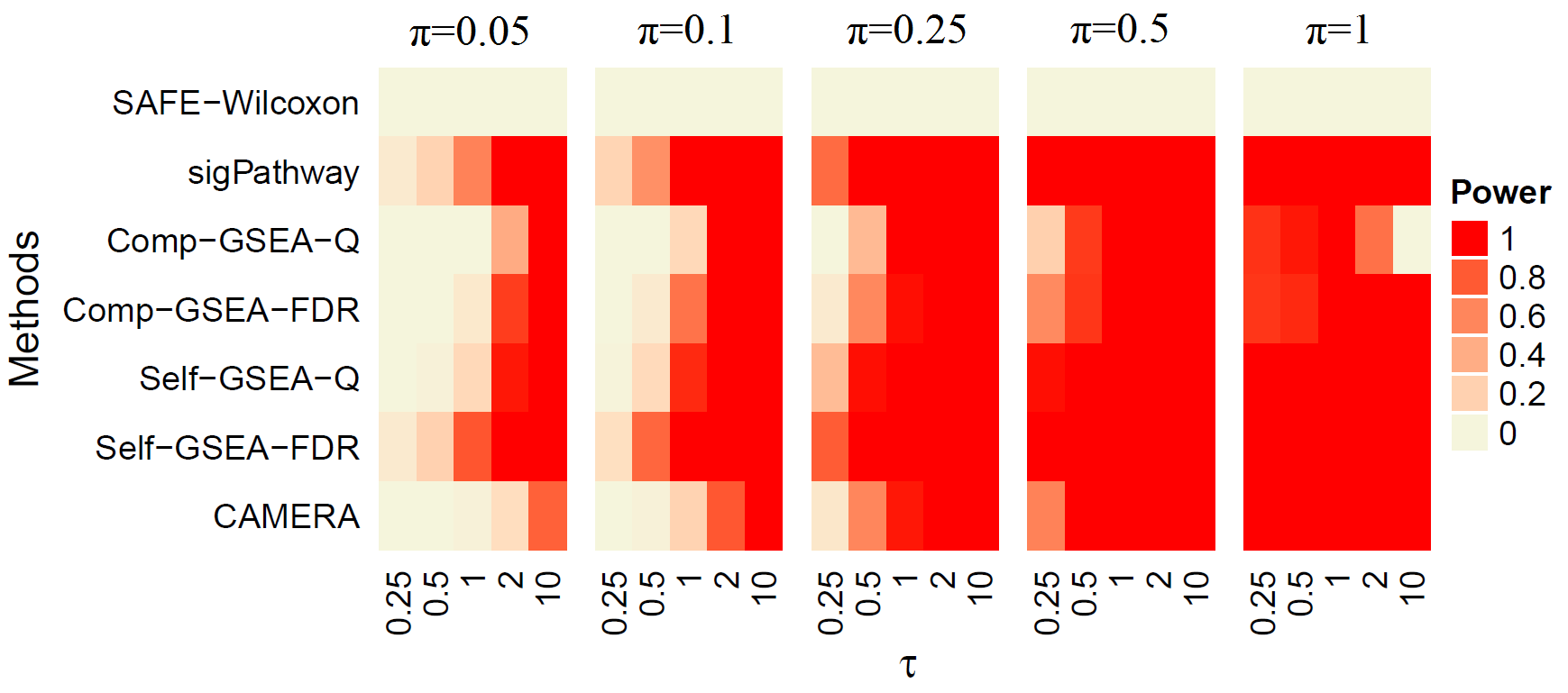


**Supplemental Figure 5:** **Power for Default Settings from Normal Brain Tissue Dataset with the GCM_FANCC pathway targeted.** Statistical power for all GSA methods tested under the default settings (including computed GSEA FDR values) from the normal brain tissue dataset with the GCM_FANCC pathway targeted for differential expression. All power values are shown as a significance value of 0.05.


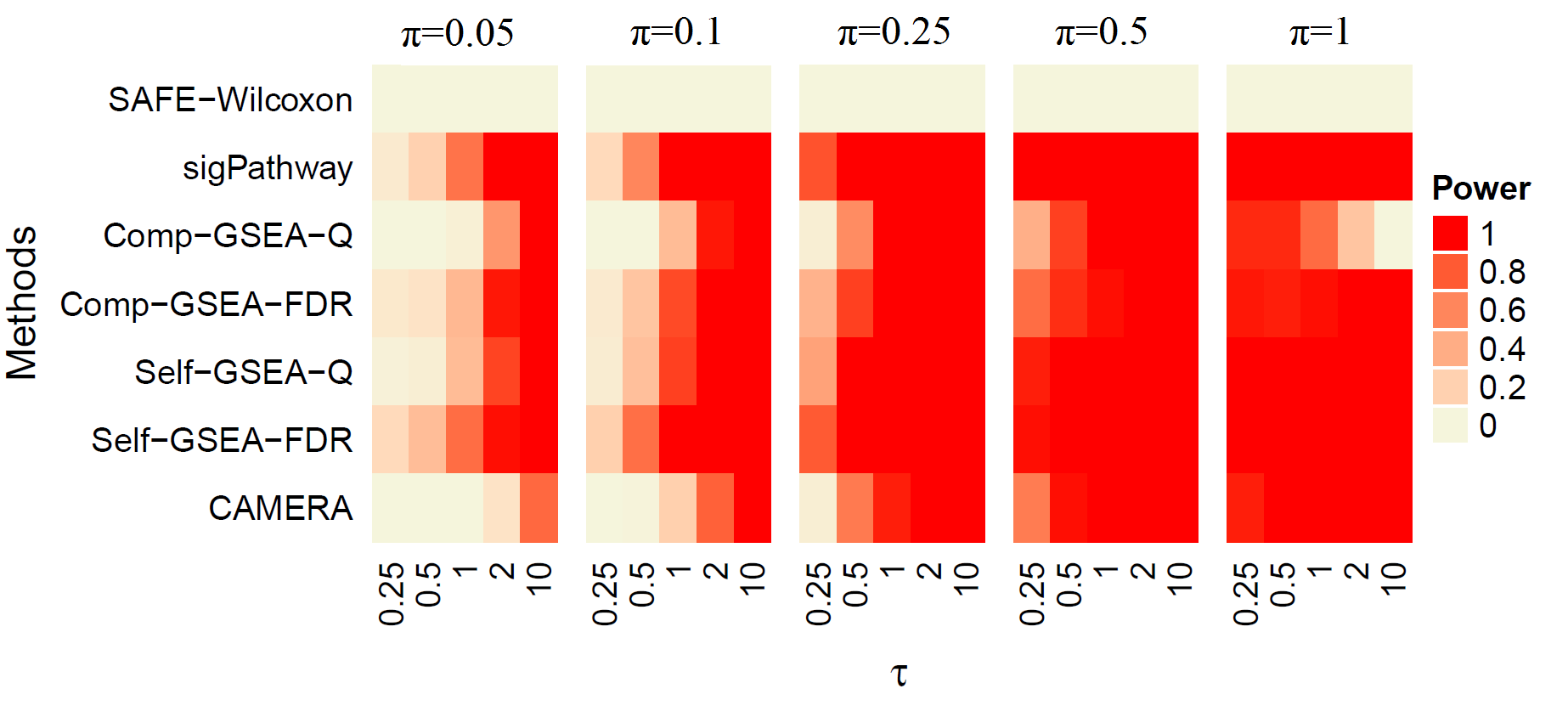


**Supplemental Figure 6:** **Power for Default Settings from Normal Brain Tissue Dataset with the metastasis pathway targeted.** Statistical power for all GSA methods tested under the default settings (including computed GSEA FDR values) from the normal brain tissue dataset with the RAMASWANY_METASTASIS_DN pathway targeted for differential expression. All power values are shown as a significance value of 0.05.

**Supplemental Table 1:** Statistical false positive rates for the three controls, negative, double negative and no signal (tau 0). All values are shown at a significance value of 0.05.

| **Method** | **Control Type** | **Mean** | **95% CI** |
| --- | --- | --- | --- |
| CAMERA | negative | 2.10E-05 | 1e-05 - 3e-05 |
| Comp GSEA | negative | 0.041675 | 0.03139 - 0.05196 |
| Self GSEA | negative | 0.049714 | 0.04821 - 0.05121 |
| aveDiff-1k | negative | 0.050651 | 0.04786 - 0.05344 |
| aveDiff-10k | negative | 0.051671 | 0.04886 - 0.05448 |
| aveDiff-boot | negative | 0.002493 | 0.00086 - 0.00413 |
| FET-1k | negative | 0.222318 | 0.21877 - 0.22587 |
| FET-10k | negative | 0.227807 | 0.22424 - 0.23138 |
| Pearson-1k | negative | 0.047051 | 0.04369 - 0.05041 |
| Pearson-10k | negative | 0.046795 | 0.0431 - 0.05049 |
| Pearson-boot | negative | 4.99E-06 | 0 - 1e-05 |
| Wilcoxon-1k | negative | 0.049958 | 0.04808 - 0.05184 |
| Wilcoxon-10k | negative | 0.050939 | 0.04904 - 0.05284 |
| Wilcoxon-boot | negative | 0.002045 | 0.00062 - 0.00347 |
| sigPathway | negative | 0.048026 | 0.04724 - 0.04881 |
| CAMERA | double negative | 0.049472 | 0.04868 - 0.05027 |
| Comp GSEA | double negative | 0.050254 | 0.04934 - 0.05117 |
| Self GSEA | double negative | 0.050387 | 0.04961 - 0.05116 |
| aveDiff-1k | double negative | 0.050639 | 0.04889 - 0.05239 |
| aveDiff-10k | double negative | 0.051587 | 0.04979 - 0.05339 |
| aveDiff-boot | double negative | 0.002147 | 0.00198 - 0.00232 |
| FET-1k | double negative | 0.188604 | 0.18492 - 0.19228 |
| FET-10k | double negative | 0.193073 | 0.18932 - 0.19683 |
| Pearson-1k | double negative | 0.050645 | 0.04806 - 0.05323 |
| Pearson-10k | double negative | 0.051696 | 0.04907 - 0.05432 |
| Pearson-boot | double negative | 0.000438 | 0.00039 - 0.00049 |
| Wilcoxon-1k | double negative | 0.050465 | 0.04875 - 0.05218 |
| Wilcoxon-10k | double negative | 0.051536 | 0.04979 - 0.05328 |
| Wilcoxon-boot | double negative | 0.0005 | 0.00044 - 0.00056 |
| sigPathway | double negative | 0.049448 | 0.04877 - 0.05012 |
| CAMERA | tau 0 | 3.89E-05 | 3e-05 - 5e-05 |
| Comp GSEA | tau 0 | 0.048615 | 0.03636 - 0.06087 |
| Self GSEA | tau 0 | 0.050618 | 0.04926 - 0.05198 |
| aveDiff-1k | tau 0 | 0.050639 | 0.04889 - 0.05239 |
| aveDiff-10k | tau 0 | 0.050673 | 0.04767 - 0.05367 |
| aveDiff-boot | tau 0 | 0.002147 | 0.00198 - 0.00232 |
| FET-1k | tau 0 | 0.215963 | 0.21285 - 0.21907 |
| FET-10k | tau 0 | 0.220485 | 0.21736 - 0.22361 |
| Pearson-1k | tau 0 | 0.047385 | 0.04415 - 0.05062 |
| Pearson-10k | tau 0 | 0.023882 | 0.01869 - 0.02907 |
| Pearson-boot | tau 0 | 0.000683 | 0.00033 - 0.00104 |
| Wilcoxon-1k | tau 0 | 0.050326 | 0.04871 - 0.05194 |
| Wilcoxon-10k | tau 0 | 0.051284 | 0.0496 - 0.05297 |
| Wilcoxon-boot | tau 0 | 0.019173 | 0.01294 - 0.0254 |
| sigPathway | tau 0 | 0.047541 | 0.04681 - 0.04827 |

**Supplemental Table 2:** Statistical power values for simulation results (for both pathways and all pairs of π and τ tested) for all methods along with GSEA alternative settings. All power values are shown at a significance value of 0.05.

| **Pathway** | **π** | **τ** | **CAMERA** | **Comp GSEA-Q** | **Comp GSEA-FDR** | **Self GSEA-Q** | **Self GSEA-FDR** | **sigPathway** | **aveDiff-1k** | **FET-1k** | **Pearson-1k** | **Wilcoxon-1k** |
| --- | --- | --- | --- | --- | --- | --- | --- | --- | --- | --- | --- | --- |
| KRAS | 0.05 | 0.25 | 0 | 0.01 | 0.25 | 0 | 0.34 | 0.91 | 0 | 0 | 0 | 0 |
| KRAS | 0.05 | 0.5 | 0 | 0.24 | 0.45 | 0.22 | 0.93 | 1 | 0 | 0 | 0 | 0 |
| KRAS | 0.05 | 1 | 0 | 0.57 | 0.87 | 0.83 | 1 | 1 | 0 | 0 | 0 | 0 |
| KRAS | 0.05 | 2 | 0 | 0.98 | 1 | 0.87 | 1 | 1 | 0 | 0 | 0 | 0 |
| KRAS | 0.05 | 10 | 0.99 | 1 | 1 | 0.86 | 1 | 1 | 0 | 0 | 0 | 0 |
| KRAS | 0.1 | 0.25 | 0 | 0.2 | 0.51 | 0.18 | 0.93 | 1 | 0 | 0 | 0 | 0 |
| KRAS | 0.1 | 0.5 | 0 | 0.55 | 0.84 | 1 | 1 | 1 | 0 | 0 | 0 | 0 |
| KRAS | 0.1 | 1 | 0 | 0.93 | 1 | 1 | 1 | 1 | 0 | 0 | 0 | 0 |
| KRAS | 0.1 | 2 | 1 | 1 | 1 | 0.86 | 1 | 1 | 0 | 0 | 0 | 0 |
| KRAS | 0.1 | 10 | 1 | 1 | 1 | 0.85 | 1 | 1 | 0 | 0 | 0 | 0 |
| KRAS | 0.25 | 0.25 | 0 | 0.95 | 0.98 | 0.99 | 0.99 | 1 | 0 | 0 | 0 | 0 |
| KRAS | 0.25 | 0.5 | 0.14 | 0.99 | 0.99 | 1 | 1 | 1 | 0 | 0 | 0 | 0 |
| KRAS | 0.25 | 1 | 1 | 1 | 1 | 1 | 1 | 1 | 0 | 0 | 0 | 0 |
| KRAS | 0.25 | 2 | 1 | 1 | 1 | 0.89 | 1 | 1 | 0 | 0 | 0 | 0 |
| KRAS | 0.25 | 10 | 1 | 1 | 1 | 0.84 | 1 | 1 | 0 | 0 | 0 | 0 |
| KRAS | 0.5 | 0.25 | 0.26 | 1 | 1 | 0.99 | 0.99 | 1 | 0 | 0.93 | 0 | 0 |
| KRAS | 0.5 | 0.5 | 1 | 1 | 1 | 1 | 1 | 1 | 0 | 0.51 | 0 | 0 |
| KRAS | 0.5 | 1 | 1 | 1 | 0.99 | 1 | 1 | 1 | 0 | 0.01 | 0 | 0 |
| KRAS | 0.5 | 2 | 1 | 1 | 1 | 0.94 | 1 | 1 | 0 | 0 | 0 | 0 |
| KRAS | 0.5 | 10 | 1 | 1 | 1 | 0.64 | 1 | 1 | 0 | 0 | 0 | 0 |
| KRAS | 1 | 0.25 | 1 | 1 | 1 | 1 | 1 | 1 | 0 | 1 | 0 | 0 |
| KRAS | 1 | 0.5 | 1 | 1 | 1 | 1 | 1 | 1 | 0 | 1 | 0 | 0 |
| KRAS | 1 | 1 | 1 | 0.49 | 1 | 1 | 1 | 1 | 0 | 1 | 0 | 0 |
| KRAS | 1 | 2 | 1 | 0 | 0.99 | 0.88 | 1 | 1 | 0 | 1 | 0 | 0 |
| KRAS | 1 | 10 | 1 | 0 | 0.94 | 0.45 | 1 | 1 | 0 | 1 | 0 | 0 |
| TGF-β | 0.05 | 0.25 | 0 | 0 | 0.12 | 0 | 0.21 | 0.08 | 0 | 0 | 0 | 0 |
| TGF-β | 0.05 | 0.5 | 0 | 0.2 | 0.33 | 0.11 | 0.5 | 0.99 | 0 | 0 | 0 | 0 |
| TGF-β | 0.05 | 1 | 0 | 0.49 | 0.73 | 0.42 | 0.99 | 1 | 0 | 0 | 0 | 0 |
| TGF-β | 0.05 | 2 | 0.79 | 0.93 | 1 | 0.92 | 1 | 1 | 0 | 0 | 0 | 0 |
| TGF-β | 0.05 | 10 | 1 | 1 | 1 | 0.92 | 1 | 1 | 0 | 0 | 0 | 0 |
| TGF-β | 0.1 | 0.25 | 0 | 0.14 | 0.35 | 0.21 | 0.68 | 1 | 0 | 0.01 | 0 | 0 |
| TGF-β | 0.1 | 0.5 | 0 | 0.47 | 0.65 | 0.89 | 1 | 1 | 0 | 0.01 | 0 | 0 |
| TGF-β | 0.1 | 1 | 0.3 | 0.89 | 0.95 | 0.92 | 1 | 1 | 0 | 0 | 0 | 0 |
| TGF-β | 0.1 | 2 | 1 | 1 | 1 | 0.87 | 1 | 1 | 0 | 0 | 0 | 0 |
| TGF-β | 0.1 | 10 | 1 | 1 | 1 | 0.88 | 1 | 1 | 0 | 0 | 0 | 0 |
| TGF-β | 0.25 | 0.25 | 0 | 0.78 | 0.96 | 1 | 1 | 1 | 0 | 0.36 | 0 | 0 |
| TGF-β | 0.25 | 0.5 | 0.87 | 1 | 0.98 | 1 | 1 | 1 | 0 | 0.18 | 0 | 0 |
| TGF-β | 0.25 | 1 | 1 | 1 | 1 | 0.94 | 1 | 1 | 0 | 0.04 | 0 | 0 |
| TGF-β | 0.25 | 2 | 1 | 1 | 1 | 0.89 | 1 | 1 | 0 | 0 | 0 | 0 |
| TGF-β | 0.25 | 10 | 1 | 1 | 1 | 0.87 | 1 | 1 | 0 | 0 | 0 | 0 |
| TGF-β | 0.5 | 0.25 | 0.62 | 1 | 1 | 1 | 1 | 1 | 0 | 1 | 0 | 0 |
| TGF-β | 0.5 | 0.5 | 1 | 1 | 1 | 1 | 1 | 1 | 0 | 1 | 0 | 0 |
| TGF-β | 0.5 | 1 | 1 | 1 | 0.99 | 0.96 | 1 | 1 | 0 | 0.54 | 0 | 0 |
| TGF-β | 0.5 | 2 | 1 | 1 | 1 | 0.86 | 1 | 1 | 0 | 0.05 | 0 | 0 |
| TGF-β | 0.5 | 10 | 1 | 1 | 1 | 0.86 | 1 | 1 | 0 | 0.01 | 0 | 0 |
| TGF-β | 1 | 0.25 | 1 | 1 | 1 | 1 | 1 | 1 | 0 | 1 | 0 | 0 |
| TGF-β | 1 | 0.5 | 1 | 1 | 1 | 1 | 1 | 1 | 0 | 1 | 0 | 0 |
| TGF-β | 1 | 1 | 1 | 0.39 | 1 | 1 | 1 | 1 | 0 | 1 | 0 | 0 |
| TGF-β | 1 | 2 | 1 | 0 | 0.98 | 0.87 | 1 | 1 | 0 | 0.76 | 0 | 0 |
| TGF-β | 1 | 10 | 1 | 0 | 0.96 | 0.83 | 1 | 1 | 0 | 0.5 | 0 | 0 |

| **Pathway** | **π** | **τ** | **aveDiff-10k** | **FET-10k** | **Pearson-10k** | **Wilcoxon-10k** | **aveDiff-boot** | **Pearson-boot** | **Wilcoxon-boot** |
| --- | --- | --- | --- | --- | --- | --- | --- | --- | --- |
| KRAS | 0.5 | 0.25 | 0 | 1 | 0 | 0 | 0.39 | 0 | 0.01 |
| KRAS | 0.5 | 0.5 | 0 | 1 | 0 | 0 | 1 | 0 | 0.01 |
| KRAS | 0.5 | 1 | 0 | 0.23 | 0 | 0 | 1 | 0 | 0.01 |
| KRAS | 0.5 | 2 | 0 | 0 | 0 | 0 | 1 | 0 | 0.01 |
| KRAS | 0.5 | 10 | 0 | 0 | 0 | 0 | 1 | 0 | 0.01 |
| KRAS | 1 | 0.25 | 0 | 1 | 0.09 | 0 | 0.64 | 0 | 0 |
| KRAS | 1 | 0.5 | 0 | 1 | 0.01 | 0 | 1 | 0 | 0 |
| KRAS | 1 | 1 | 0 | 1 | 0 | 0 | 1 | 0 | 0 |
| KRAS | 1 | 2 | 0 | 1 | 0 | 0 | 1 | 0 | 0 |
| KRAS | 1 | 10 | 0 | 1 | 0 | 0 | 1 | 0 | 0 |
| TGF-Beta | 0.5 | 0.25 | 0 | 1 | 0 | 0 | 0.55 | 0 | 0.01 |
| TGF-Beta | 0.5 | 0.5 | 0 | 1 | 0 | 0 | 1 | 0 | 0.01 |
| TGF-Beta | 0.5 | 1 | 0 | 0.77 | 0 | 0 | 1 | 0 | 0.01 |
| TGF-Beta | 0.5 | 2 | 0 | 0.09 | 0 | 0 | 1 | 0 | 0.01 |
| TGF-Beta | 0.5 | 10 | 0 | 0.04 | 0 | 0 | 1 | 0 | 0.01 |
| TGF-Beta | 1 | 0.25 | 0 | 1 | 0.14 | 0 | 0.73 | 0 | 0.17 |
| TGF-Beta | 1 | 0.5 | 0 | 1 | 0.02 | 0 | 1 | 0 | 0.23 |
| TGF-Beta | 1 | 1 | 0 | 1 | 0 | 0 | 1 | 0 | 0.23 |
| TGF-Beta | 1 | 2 | 0 | 0.96 | 0 | 0 | 1 | 0 | 0.23 |
| TGF-Beta | 1 | 10 | 0 | 0.93 | 0 | 0 | 1 | 0 | 0.23 |

**Supplemental Table 3:** Statistical power values for simulation results (for both pathways and all pairs of π and τ tested) with alternative settings for SAFE. All power values are shown at a significance value of 0.05.


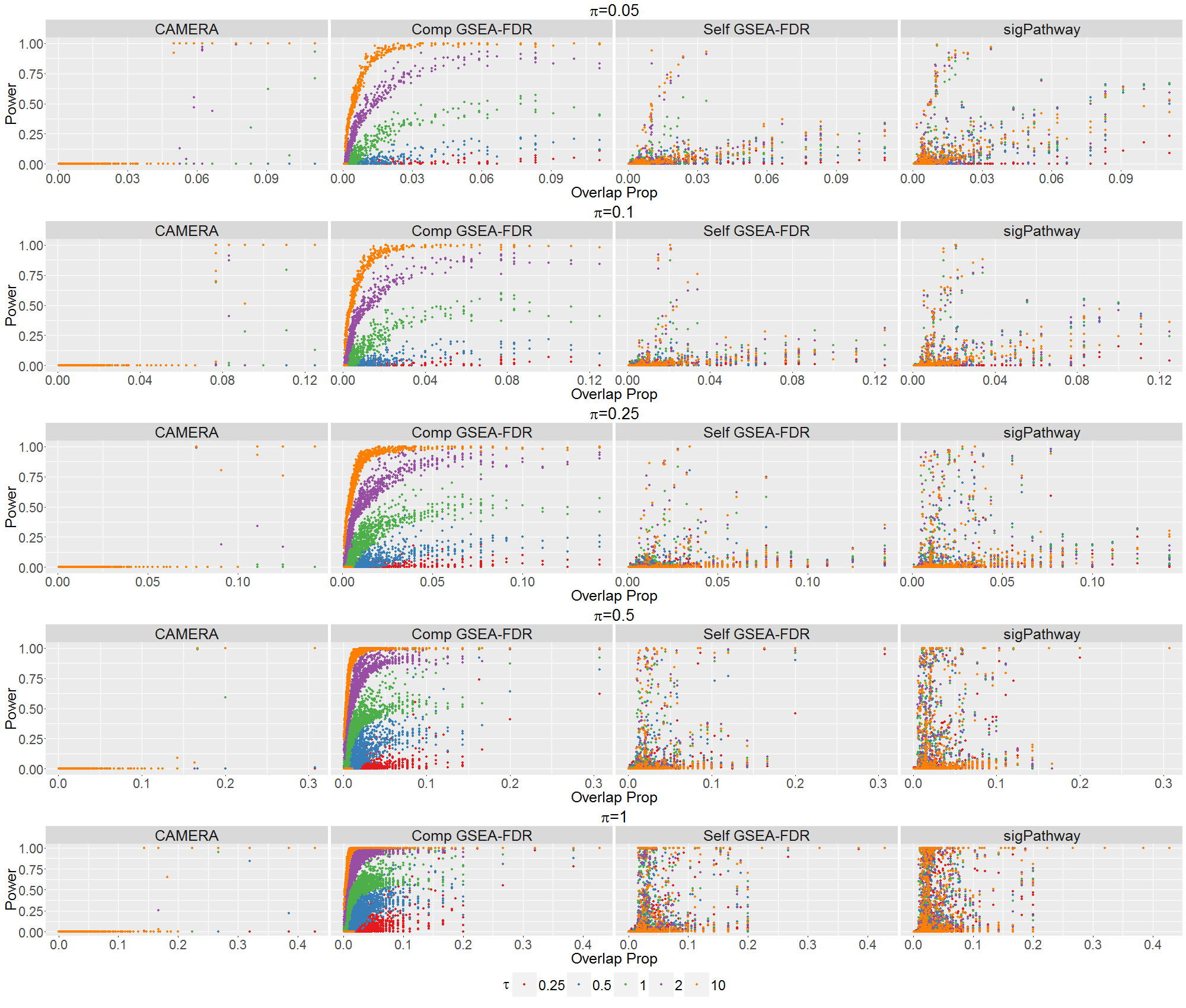


**Supplemental Figure 7: Power of Overlap Pathways for all effect sizes for KRAS Pathway.** Statistical power for results with recommended methods for overlap pathways for experiments with the KRAS pathway differentially expressed. The recommended methods include CAMERA, GSEA with user defined FDR q-value and sigPathway. The x-axis denotes the proportion of the pathway that overlaps with the KRAS pathway. Each point in the graph represents one of these overlapping pathways. All power values are shown as a significance value of 0.05.


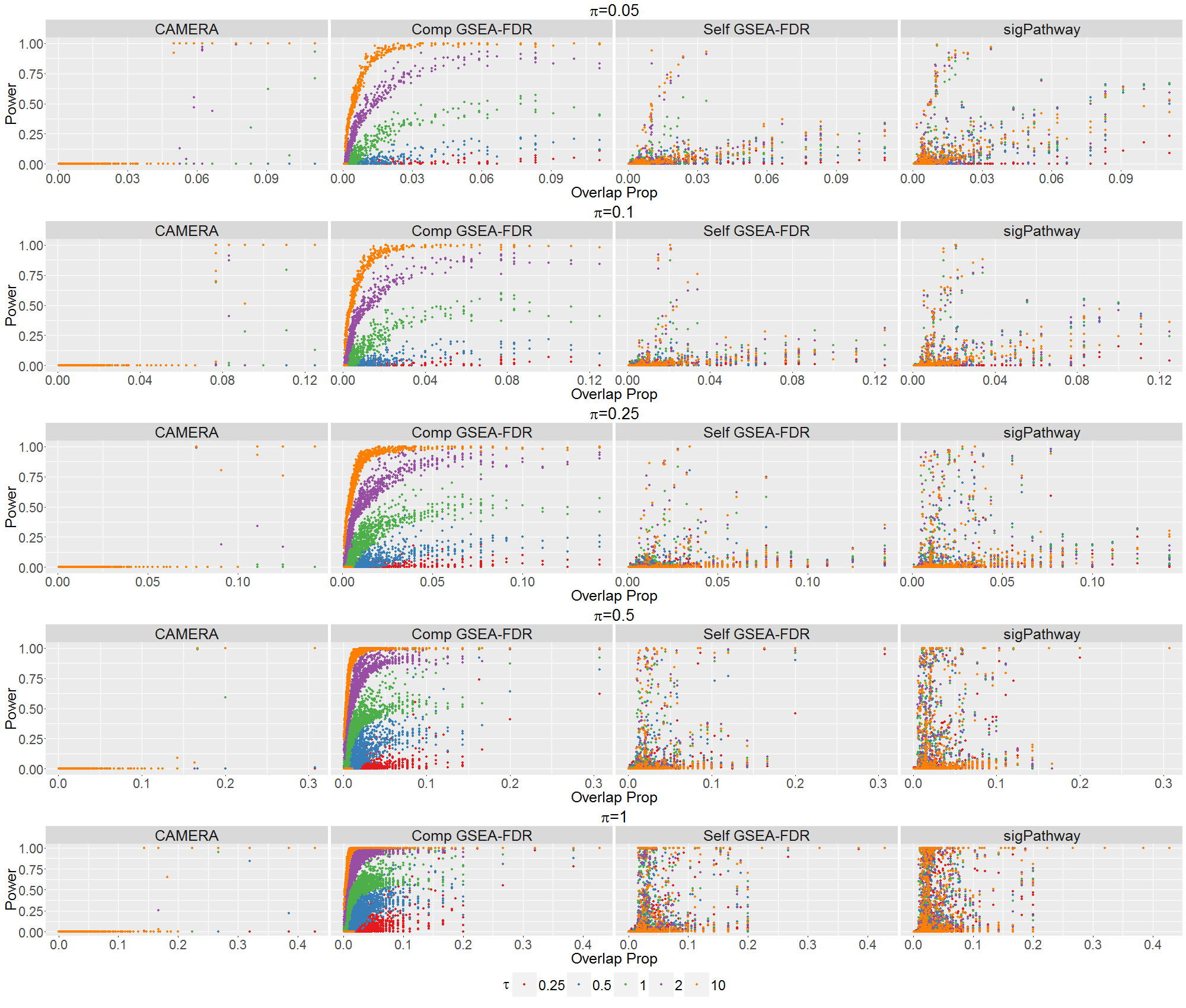


**Supplemental Figure 8:** **Power of Overlap Pathways for all effect sizes for TGF-β Pathway.** *Statistical power for results with recommended methods for overlap pathways for experiments with the TGF-*β *pathway differentially expressed. The recommended methods include CAMERA, GSEA with user defined FDR q-value and sigPathway. The x-axis denotes the proportion of the pathway that overlaps with the TGF-*β *pathway. Each point in the graph represents one of these overlapping pathways. All power values are shown as a significance value of 0.05.*
